# Visual-Numeric Endometriosis Scoring System (VNESS) for mapping surgical findings: A validation study

**DOI:** 10.52054/FVVO.16.4.051

**Published:** 2024-12-27

**Authors:** S Khazali, B Mondelli, K Fleischer, A Bachi, M Adamczyk, N Lemos, H Krentel, A Vashisht, A Abdalla, A Mohazzab, G Delanerolle, M Possover, R Padmehr, K Shadjoo, G Moawad, T Lee, E Saridogan

**Affiliations:** HCA the Lister Hospital- Centre for Endometriosis and Minimally Invasive Gynaecology (CEMIG London). London, United Kingdom; Royal Holloway, University of London, London, United Kingdom; Ashford and St Peter’s Hospitals NHS Foundation Trust, Chertsey, Surrey, United Kingdom; Royal Surrey County Hospital, Guildford, Surrey, United Kingdom; Department of Obstetrics and Gynecology, Mount Sinai Hospital, Toronto, Canada; Department of Gynecology, Obstetrics and Gynecological Oncology, Bethesda Hospital, Duisburg, Academic Teaching Hospital, Germany; University College London Hospitals NHS Foundation Trust, London, United Kingdom; Consultant in Obstetrics & Gynaecology, London, United Kingdom; School of public health, Iran University of Medical Sciences, Tehran, Iran; Southern Health NHS Foundation Trust, United Kingdom; University of Birmingham, United Kingdom; Possover International Medical Center AG, Zurich, Switzerland; Department of Obstetrics and Gynaecology, Avicenna Research Institute, Tehran, Iran, Islamic Republic of Iran; Reproductive Biotechnology Research Center, Avicenna Research Institute, ACECR, Tehran, Iran, Islamic Republic of Iran; Department of Obstetrics and Gynecology, The George Washington University Hospital, Washington DC, USA; Department of Obstetrics, Gynecology, and Reproductive Sciences, University of Pittsburgh School of Medicine, UPMC Magee-Womens Hospital, Pittsburgh, USA; * These authors contributed equally to this work.

**Keywords:** Endometriosis, scoring system, endometriosis classification, VNESS

## Abstract

**Background:**

Several endometriosis classification systems have been proposed and published but the search for a universal language that communicates the complexity, laterality and severity of this disease continues. The authors introduce the Visual-Numeric Endometriosis Scoring System. VNESS is a novel system for describing surgical findings in each compartment of the pelvis in a way that is simple to use, visually intuitive and mirrors a laparoscopic image of the pelvis.

**Objective:**

The aim of this study was to assess inter-rater reliability for components of VNESS.

**Materials and Methods:**

The project took the format of a validation study using short surgical laparoscopic video clips. Anonymised video clips of endometriosis procedures were scored by 50 Gynaecologists of varying levels of experience from 12 different countries. The clips were collated from a series of procedures performed between 2012 and 2022. Each participant scored 93 short surgical clips using VNESS. 4650 scores were compared against a reference score and analysis was performed to assess inter-rater reliability.

**Main outcome measures:**

The outcome measures were percentage agreement between given and reference scores, as well as intra-class correlation coefficients (ICC), Cohen Kappa and Quadratic Weighted Kappa Coefficients calculated to evaluate inter-rater reliability.

**Results:**

The highest and lowest percentage agreement with the reference score was seen in VNESS 4 (full thickness disease, 97% perfect agreement) and VNESS 1 (superficial disease, 53% perfect agreement) respectively. The intraclass correlation coefficient showed strong inter-rater reliability for all VNESS compartments except the vagina.

**Conclusions:**

This study suggests that VNESS has excellent reliability between observers. Correlation is stronger with more severe disease.

## Introduction

Over twenty published classification/staging systems have been proposed over the last four decades. Despite this, the search for a universally accepted language for communicating the complexity and severity of this enigmatic disease continues. These systems have recently been evaluated and reviewed (International Working Group of AAGL, ESGE, ESHRE and WES et al., 2021).

The revised American Society for Reproductive Medicine (rASRM) system is still the most widely used (International Working Group of AAGL, ESGE, ESHRE and WES et al., 2022) even though most experts agree that the system is not fit for purpose, particularly for description of deep endometriosis (Haas et al., 2013b; Haas et al., 2013c; Keckstein and Hudelist, 2021; Padmehr et al., 2021; Hudelist et al., 2021; Montanari et al., 2022b).

ENZIAN, first published in 2005, pays particular attention to retroperitoneal and deep disease (Tuttlies et al., 2005) and its latest version, #ENZIAN simplifies the original system, whilst also includes the peritoneum, ovaries and fallopian tubes, and provides comprehensive definition of deep endometriosis in transvaginal ultrasound, magnetic resonance (MR) imaging and surgery (Keckstein et al., 2021). Some of the benefits of the #ENZIAN system are that it provides mapping of deep endometriosis (Keckstein and Hudelist, 2021; Hudelist et al., 2021; Montanari et al., 2022b), there is correlation and reproducibility between imaging results with surgical findings in most of the compartments (Di Giovanni et al., 2021; Montanari et al., 2022a; Harth et al., 2023; Keckstein and Hoopmann, 2023; Maciel et al., 2023) and there is correlation between disease severity and surgical complexity/complications with both the old ENZIAN and newer #ENZIAN (Haas et al., 2013 (a); Haas et al., 2013 (b); Poupon et al., 2019; Aas-Eng et al., 2022). #ENZIAN addresses the limitations of the original ENZIAN system (Haas et al., 2013a); it is comprehensive and aims to map endometriosis both surgically and radiologically so that there can be a clear understanding of the location of the disease and what procedures, as well as their associated risks, would be required to manage it.

In 2021, AAGL introduced a staging system; a weighted score is allocated based on expert-derived surgical complexity ratings for each anatomical site (Abrao et al., 2021). It is straightforward for patients to understand and correlates with surgical complexity (Abrao et al., 2021). This was an evidence-based attempt at incorporating the complexity of surgery but the final staging of the AAGL system communicates limited information about the location and laterality of the disease (unless location is specified also), uses arbitrary size brackets and requires an application to calculate the final score.

Despite recent attempts with the #ENZIAN and AAGL systems to address the limitations of rASRM, there is still no universally accepted system that maps all locations of the disease.

VNESS was conceptualised in 2014 in an attempt to find a way to record and communicate the surgical findings and the complexity of surgery being undertaken in a very busy tertiary referral centre for endometriosis.

The aim was to create a system that was precise but also easy to use and understand. A system similar to the POP-Q (Pelvic Organ Prolapse Quantification system), but for endometriosis. It was devised to closely mirror the pelvic survey at laparoscopy. Not only could this visual representation be easy to understand for both patient and clinician, but also encompasses the laterality of the disease.

The system started with a scale of 0 to 6 (6 representing full-thickness invasion into surrounding structures. There was no score of 5 to reduce the risk of overlap and to accentuate the clinical importance of tissue invasion). The first version of VNESS had 8 anatomical compartments and did not separate the vagina from the rectum.

In 2015, a validation study on this version of VNESS was done as part of a Master’s degree thesis, using 5 scorers scoring 63 videos, each video showing all compartments of the pelvis, showed excellent inter and intra-observer validity.

Following consultation with multiple international experts, VNESS was refined and simplified to include only 4 levels of severity. A further compartment was added in order to distinguish vaginal involvement from rectal disease. Once the final version was determined, SK and BM devised an appropriate preliminary study to best assess the reproducibility and validity of the VNESS classification system.

The aim of the study was to determine if a VNESS score could be reproducible between observers, not to assess non-inferiority nor superiority to other classification systems.

## Materials and Methods

### Visual Numeric Endometriosis Scoring System (VNESS) Description

VNESS consists of nine numbers, each representing one compartment of the pelvis. The numbers are written from left to right, with the first number representing the left adnexa and the last number on the far right representing the right adnexa. The three numbers in the centre correspond to the central pelvic structures. The anatomical compartments, from left to right, are described as follows:

Left adnexa (LADN), left pelvic sidewall (LPSW), left uterosacral ligament (LUSL), uterovesical fold (UVF), Vagina and rectovaginal space (VAG), pouch of Douglas and Rectum (RECT), right uterosacral ligament (RUSL), right pelvic sidewall (RPSW) and right adnexa (RADN).

**Figure 1:**
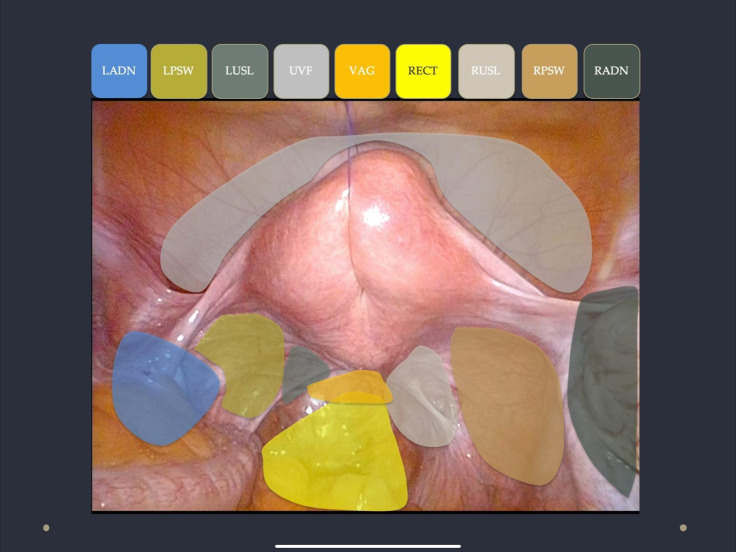
Anatomical compartments represented in the VNESS classification system.

These compartments are shown in Figure 1.

A disease severity score, ranging from 0 to 4, is allocated for each anatomical compartment. The severity scale is described as follows:

0. No macroscopic evidence of endometriosis
1. Superficial endometriosis
2. Deep endometriosis with no adhesions or with filmy adhesions to surrounding structures
3. Deep endometriosis with dense adhesions to surrounding structures
4. Deep endometriosis invading into surrounding structures

The order in which these numbers are written aids in visualising the severity of the disease in each pelvic compartment mirroring the pelvic survey during a diagnostic laparoscopy.

The intention is that a clinician would be able to visualise the severity of disease in each compartment by seeing VNESS, as if they were looking at a laparoscopic image of the pelvis.

**Figure 2:**
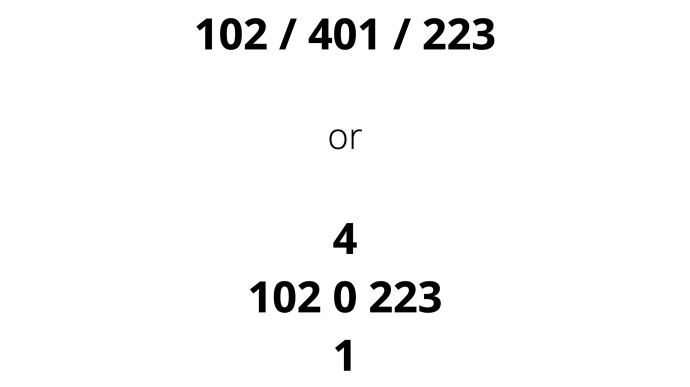
How to document a VNESS Score.

VNESS can be written in a linear manner with a slash separating the right, central and left compartments or in cross-cross-shaped format with the central compartment numbers written on top of each other. The cross format would be more suitable for handwritten notes and facilitates visualisation of the disease further (Figure 2).

Extra pelvic endometriosis, adenomyosis or other findings are written as free text after the VNESS score (for example 334/034/322 Appendix 4 Diaphragm 1).

Ideally, there should be a comprehensive preoperative assessment and radiological investigation in order to provide the most appropriate surgery. There are still scenarios, however, where clinicians perform a diagnostic laparoscopy and may not be certain if there is invasion into surrounding viscera. In these circumstances, where invasion remains queried, then a “?” is put in place of a score for the uncertain compartment. For example, 10?/401/223 would indicate that the left uterosacral ligament cannot be assessed for disease severity. It is not the authors’ intention to insinuate that a clinician should feel comfortable performing a procedure if the extent and depth of infiltration is not known, rather the “?” should be a prompt to either perform further investigation to confirm the depth of infiltration or as signposting when a generalist may be referring on to endometriosis specialist that a particular severity is suspected but not confirmed and further evaluation is required.

If a compartment is absent, for example following adnexectomy, this can be denoted with an ‘X’. X11/111/11X would indicate that both fallopian tubes and ovaries were absent in a pelvis with widespread superficial disease.

## Materials and Methods

### Visual Numeric Endometriosis Scoring System (VNESS) Description

VNESS consists of nine numbers, each representing one compartment of the pelvis. The numbers are written from left to right, with the first number representing the left adnexa and the last number on the far right representing the right adnexa. The three numbers in the centre correspond to the central pelvic structures. The anatomical compartments, from left to right, are described as follows:

Left adnexa (LADN), left pelvic sidewall (LPSW), left uterosacral ligament (LUSL), uterovesical fold (UVF), Vagina and rectovaginal space (VAG), pouch of Douglas and Rectum (RECT), right uterosacral ligament (RUSL), right pelvic sidewall (RPSW) and right adnexa (RADN).

These compartments are shown in Figure 1.

A disease severity score, ranging from 0 to 4, is allocated for each anatomical compartment. The severity scale is described as follows:

0. No macroscopic evidence of endometriosis
1. Superficial endometriosis
2. Deep endometriosis with no adhesions or with filmy adhesions to surrounding structures
3. Deep endometriosis with dense adhesions to surrounding structures
4. Deep endometriosis invading into surrounding structures

The order in which these numbers are written aids in visualising the severity of the disease in each pelvic compartment mirroring the pelvic survey during a diagnostic laparoscopy.

The intention is that a clinician would be able to visualise the severity of disease in each compartment by seeing VNESS, as if they were looking at a laparoscopic image of the pelvis.

VNESS can be written in a linear manner with a slash separating the right, central and left compartments or in cross-cross-shaped format with the central compartment numbers written on top of each other. The cross format would be more suitable for handwritten notes and facilitates visualisation of the disease further (Figure 2).

Extra pelvic endometriosis, adenomyosis or other findings are written as free text after the VNESS score (for example 334/034/322 Appendix 4 Diaphragm 1).

Ideally, there should be a comprehensive preoperative assessment and radiological investigation in order to provide the most appropriate surgery. There are still scenarios, however, where clinicians perform a diagnostic laparoscopy and may not be certain if there is invasion into surrounding viscera. In these circumstances, where invasion remains queried, then a “?” is put in place of a score for the uncertain compartment. For example, 10?/401/223 would indicate that the left uterosacral ligament cannot be assessed for disease severity. It is not the authors’ intention to insinuate that a clinician should feel comfortable performing a procedure if the extent and depth of infiltration is not known, rather the “?” should be a prompt to either perform further investigation to confirm the depth of infiltration or as signposting when a generalist may be referring on to endometriosis specialist that a particular severity is suspected but not confirmed and further evaluation is required.

If a compartment is absent, for example following adnexectomy, this can be denoted with an ‘X’. X11/111/11X would indicate that both fallopian tubes and ovaries were absent in a pelvis with widespread superficial disease.

### Study Design

This inter-rater reliability study took the form of an online questionnaire whereby participants answered background questions and then reviewed a short video presentation describing/explaining VNESS. Participants then reviewed several video clips showing an example of endometriosis and allocated a VNESS score according to the severity.

Ninety-three short laparoscopic video clips showing endometriosis in different pelvic compartments were selected. Each clip focused on only one compartment of the pelvis. The clip lengths ranged between 4 and 32 seconds (Mean=16.7s, Median=16s).

Each clip was scored by the principal investigator, prior to commencement of the study, and saved to be used as the “reference score” (VNESS1=17, VNESS2=32, VNESS3=27, VNESS4=17). There were no clips of VNESS 0 included, as the aim of the study wasn’t to assess the ability of scorers to recognise if endometriosis was present but to assess the severity of the disease in the clip provided.

The clips were embedded into an online data collection form as separate questions in no particular order. The form included questions assessing the experience and familiarity of the scorers with endometriosis and collected feedback regarding the ease of use as well as general comments on VNESS. The background questions were used as a guide to gauge a participant’s experience and percentage of their clinical time spent in the care of those with endometriosis.

Two hundred and ten (n=210) gynaecologists or trainees were invited to take part in the study. The criteria for inclusion as a scorer were self- claimed familiarity with endometriosis surgery and willingness to dedicate around one hour of focused time for scoring. An invitation was sent to a) all centre leads within the British Society of Gynaecological Endoscopy (BSGE) accredited Endometriosis centres b) those who responded to an open invitation on the BSGE Facebook page c) a network of colleagues known to the authors.

Of 210 invited, 54 initiated the scoring process. 50 completed the process and scored all 93 videos. All complete responses were included in the study.

Responders were asked to score the severity of the endometriosis from VNESS 1 to VNESS 4. The participants were aware of what compartment was being asked to be scored. For example, if the pouch of Douglas was displayed, this was made clear in the video clip.

Examples of each VNESS severity level are provided in Appendices 1-4. These are screenshots taken from sixteen of the ninety-three video clips embedded into the survey and provide context into what the participants were asked to evaluate. Each video clip shows endometriosis prior to and during excision.

The survey was collated, and statistical analysis was performed to compare scores between scorers and the intended score as well as amongst all scorers.

### Statistical Analysis

All categorical and quantitative variables were reported as frequency (percentage) and mean (SD), respectively. The statistical analysis was performed to compare scores between scorers and the intended score as well as amongst all scorers.

Chi-squared test was used to compare categorical variables, and the independent t-test was used for quantitative variables in the subgroup analysis of baseline information.

Agreement between reference scores and observed scorers’ score was reported across two levels, absolute agreement (where the participant’s score was the same as the reference score) and partial agreement (+/- 1 score from reference score), and represented descriptively as a percentage of agreement. These agreements were reported for all compartments pooled and for each compartment separately.

In addition to the statistical assessment of the agreement, intra-class correlation coefficients (ICC) were calculated using Two-Way Mixed- Effects, with consistency definition to adjust the agreement between multiple selected rates who rate a unique set of subjects. In other terms, ICC assesses clusters of data, in this example the scores, and determines if the similarities are due to chance. The reliability of the scores varies between compartments. An ICC of <0.5 is considered poor, 0.5-0.75 moderate, 0.75-0.9 good and >0.9 excellent (Koo and Li, 2016).

Cohen and Quadratic Weighted Kappa were used to evaluate the absolute and partial agreements between reference and observed score respectively. They can be interpreted based on the Kappa coefficient as below: >0.90 Almost Perfect, 0.80-0.90=Strong 0.60-0.79=Moderate, 0.40- 0.59=Weak, 0.21-0.39 = Minimal (McHugh, 2012).

The agreement plots were drawn using Analyze-It Microsoft excel Add-in to generate the visual representation of the agreements. Statistical analysis was performed using IBM SPSS version 22 and STATA version 16. The level of significance was considered as 0.05.

## Results

Fifty scorers across twelve countries and forty- five units scored ninety-three (n=93) clips using the VNESS system (26 from the United Kingdom, 7 from Brazil, 5 from Iran, 3 from Canada, 2 from the USA, and one each from Italy, Mexico, Netherlands, South Africa, Germany, Greece and Switzerland). Eight out of 50 scorers were speciality trainees with a special interest in endometriosis.

To gauge familiarity and expertise in endometriosis surgery, the responders were asked about the proportion of their workload dedicated to the treatment of endometriosis and the complexity level of surgery such as the number of segmental bowel resections they have performed either as primary surgeons or jointly with a colorectal surgeon during multidisciplinary surgery. Trainee responders were asked the number of shaves they had performed. Appendices 5 and 6 summarise the results. It should be noted that a segmental bowel resection alone should not be the sole determinant of the level of complexity in a surgeon or a unit’s workload but does provide a surrogate indicator of the level of severity a participant may encounter in their day-to-day practice.

Results were collected and analysed as a collection of correct/incorrect scores. Therefore, a single participant’s scores were not assessed as an individual but grouped with all responses. The participants’ collective responses are then compared against the reference score allocated by SK.

The highest and lowest percentage agreement with the reference score was seen in VNESS 4 and VNESS 1 respectively. Seventeen (n=17) clips of VNESS 4 were shown. Of 850 scores received for these videos, (17x50=850), 825 (97%) were in perfect agreement with the reference score, and the rest (3%) scored the videos at VNESS 3.

Seventeen (n=17) videos of VNESS 1 endometriosis were shown. Fifty-three percents (53%) of the answers (450 out of 850 answers) perfectly matched the reference score. Thirty-eight percent (323 out of 850 scores) gave a VNESS 2 score to videos with a reference VNESS score of 1.

The percentage of perfect agreement for VNESS 2 (32 clips) and VNESS 3 (27 clips) was 69% and 82% respectively.

**Figure 3:**
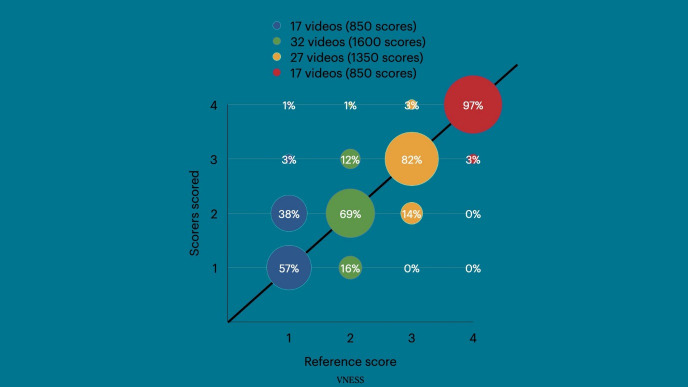
Percentage of agreement between given and reference scores for each VNESS compartment.

These results are displayed in Figure 3.

The percentage of agreement between the given scores and the intended score for videos for each pelvic compartment is summarised in Table I (Agreement Plots for the different pelvic compartments are located in the Appendices 7-12).

**Table I:** Percentage of agreement between the given scores and the intended score for videos for each pelvic compartment.

Compartment	Number of Videos	Number of videos for each reference Score				Total Scored	Correct scores	Percentage of agreement +/- 1	Percentage of perfect agreement
		1	2	3	4				
Adnexa	19	2	5	12	0	950	815	100%	85.8%
Pelvic side wall	11	4	5	1	1	550	389	98.72%	70.7%
Rectum	15	3	1	6	5	750	631	99.86%	84.1%
Uterosacral ligament	15	4	7	4	0	750	497	96.8%	66.3%
Uterovesical fold	22	4	14	1	3	1100	772	99.09%	70.2%
Vagina and rectovaginal septum	11	0	0	3	8	550	482	100%	87.6%
Total	93	17	32	27	17	4650	3586	99.9%	77.1%

As aforementioned, ICC can be used to measure reproducibility between observers. Table II shows the ICC for all 4650 scores was 0.905 (P<0.001). The vaginal compartment shows a poor ICC compared to the others; this may represent a statistical anomaly as percentage agreement was high for this compartment (87.6% perfect agreement with the reference score). This may be explained by the high reference severity score for the videos demonstrating this compartment (Table II).

**Table II:** Total ICC and ICC for each VNESS Compartment.

Compartment	ICC Average measures (Two mixed-way, Consistency)	95% Confidence Interval	P-Value
Adnexa	0.664	0.512-0.785	<0.001
Pelvic side wall	0.721	0.591-0.823	<0.001
Rectum	0.968	0.953-0.979	<0.001
Uterosacral ligament	0.810	0.723-0.879	<0.001
Uterovesical fold	0.804	0.715-0.874	<0.001
Vagina	0.308	-0.015-561	0.03
Total	0.905	0.863-0.939	<0.001

The results of the Cohen kappa and quadratic weighted kappa are displayed in Table III. These have been calculated using the standard score against the responses. Weighted kappa adjusts for the range of disagreement between observers. Since there was a high proportion of responses with only a small magnitude of difference to the reference score, the total quadratic weighted kappa coefficient is 0.864 which would be classified as strong and the Cohen Kappa is 0.678, classified as moderate (McHugh, 2012).

**Table III:** Total Cohen Kappa/Quadratic Weighted Kappa scores and scores for each VNESS compartment.

		Total	Adnexa	Pelvic Sidewall	Rectum	USL	UVF	Vagina
Cohen Kappa	Coefficient	0.678	0.720	0.554	0.780	0.410	0.521	0.712
	Wald 95% confidence Interval	0.661 - 0.695	0.680 - 0.761	0.493 - 0.615	0.745 - 0.815	0.356 - 0.464	0.477 - 0.564	0.658 - 0.767
Weighted Kappa	Coefficient	0.864	0.828	0.803	0.920	0.551	0.809	0.797
	Wald 95% confidence Interval	0.85 - 0.873	0.798 - 0.857	0.764 - 0.842	0.904 - 0.936	0.498 - 0.603	0.784 - 0.834	0.767 - 0.827

## Discussion

**Figure 4:**
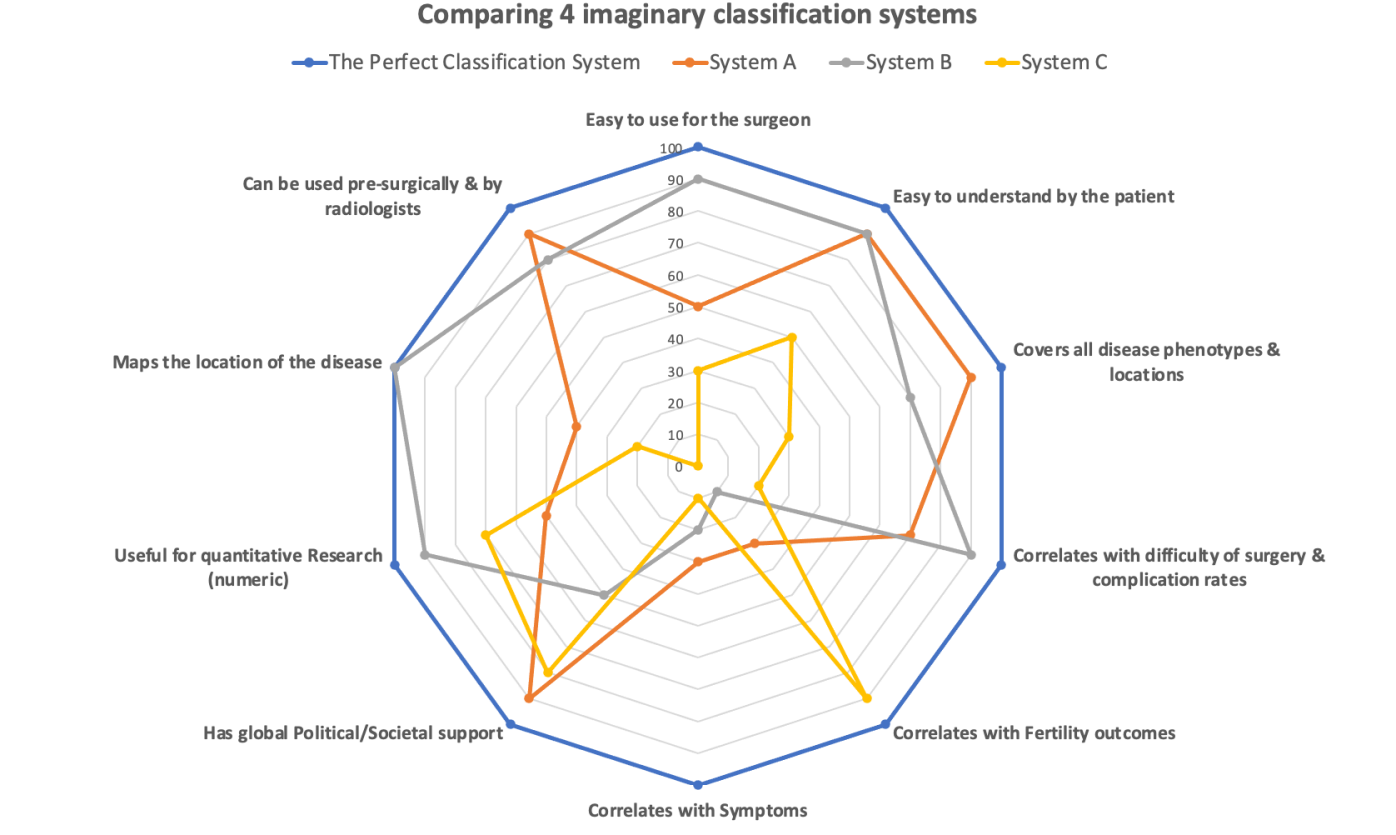
Percentage of agreement between given and reference scores for each VNESS compartment.

An “ideal” system for classification and staging of endometriosis is more likely to organically evolve from a combination of multiple systems, rather than the growth or promotion of a single system. The nature of the disease makes it very difficult, if not impossible, to design an all-encompassing system that fulfils all requirement of a perfect system. We previously made this point in an editorial article (Khazali and Saridogan, 2021) (Figure 4).

Without further future comparative research, it is not possible to comment on where VNESS fits in terms of the current classification systems. The authors believe that the use of technology can and will facilitate the calculation of multiple staging systems from “raw surgical data”. This will eliminate the need to choose between systems and in time will lead to a system where the best parts of all proposed systems can be put together to generate a universal language for communication. It is for these reasons that we propose that VNESS be used in conjunction with, rather than in place of, other validated systems. We believe VNESS can fill the gaps in areas not sufficiently addressed by other systems such as the ability to map the location and laterality of the disease and being visually intuitive and easy to understand and communicate.

### Main Findings

This study indicates that VNESS has good inter- observer reproducibility. Correlation is stronger with more severe disease.

The weakest correlation was noted with less severe disease. This weakness was more prominent where the intended score was 1 (17 video clips). These videos were scored correctly in 57% but 38% of scorers scored these videos as 2, meaning the scorers judged the disease to be deep. This is not surprising, given the fact that the scorers did not have the benefit of tactile feedback which could have aided better discretion between superficial and deep disease. Furthermore, the definition of “superficial” and “deep” endometriosis is still not universally agreed amongst endometriosis experts. In the authors’ opinion, the historical definition that describes deep endometriosis as a disease with a depth >5 mm is poorly applicable in clinical practice. We consider any endometriosis that goes beyond the fine peritoneal layer as deep.

In designing VNESS, there have been a number of deliberate inclusions and exclusions. These considerations aim to strike a balance between ease of use and being exhaustive but without becoming restrictive.

### VNESS as a surgical mapping system

VNESS does not have any arbitrary size brackets for endometriotic lesions or for endometriomas. It is the authors’ opinion that, with some exceptions, the size of an endometriotic nodule should not be the sole guide to the complexity of the disease. For example, a small nodule that involves the full thickness of the ureter is likely to be more complex to excise compared to a large nodule involving the dome of the bladder. There are of course situations, for example, rectal nodules, in which removal of a larger lesion may require a more invasive intervention. The data available on the management of bowel endometriosis according to size, however, still doesn’t have a consensus agreement. With some papers pointing to specific criteria for segmental bowel resection (Malzoni et al., 2020; Wojtaszewska et al., 2022). Whilst others advocate managing even large lesions with a potentially more conservative approach (Donnez, 2021; Roman et al., 2023).

Extrapelvic endometriosis and specific anatomical structures are not categorised within VNESS. Instead, the surgeon can add any additional information as a note (e.g. 320/100/221-Diaphragm 1). We believe that the inclusion of all extrapelvic structures within the body of the scoring system can detract from the simplicity of the system.

One of the strengths of VNESS is that the numerical/compartment system leads itself to statistical analysis and will provide a platform for further research. This advantage, along with the ability to map the disease, can complement other systems which include extrapelvic locations and size of nodules, such as #ENZIAN, and be used alongside them to form a more complete picture of the surgical findings.

Thus, the results from this preliminary study suggest that the VNESS tool is reproducible and correlated with identifying and reporting disease severity. Future planned research intends to explore if there is a correlation between VNESS score with symptoms, pre-operative imaging and surgical complexity.

### Limitations of this study

Whilst this study shows excellent inter-rater reliability, it does not assess the ability of the surgeon to allocate scores to correct compartments. In practice, it is likely that there will be some overlap between adjacent compartments leading to these compartments being scored similarly as there is no sharp and clear anatomical border for example between the pelvic sidewall and the uterosacral ligament. The scope of this study was to demonstrate that the initial success of VNESS. We aim to assess any correlations between VNSS with surgical complexity, complications and pre- operative imaging in the future.

The use of video clips has some limitations in comparison to real-life surgery. With the benefit of tactile feedback and a longer time to assess the severity of each compartment, it is likely that this study underestimates the reliability of scoring, especially for less severe disease.

Clips with VNESS score of 4 in VAG, RECT and UVF compartments showed the procedure to excise these lesions (partial vaginectomy, discoid or segmental bowel resection or partial cystectomy respectively). This would have made the intended score obvious to the scorer. Whilst this could be considered a selection bias, it is important to keep in mind that the aim of this study was not to test the ability of the scorers to “guessing” the depth of invasion or to assess their decision-making ability, but to evaluate if VNESS offers a reliable language for communicating the surgical findings.

Equally, the study has asked participants to evaluate a single compartment at a time, and this has been analysed accordingly, rather than assessing and interpreting universal VNESS score. This may retract from the reliability of VNESS as a whole, however, if a clinician is documenting a procedure, they would typically do this by each compartment at a time, so this study does not deviate significantly from ‘real-world’ practice.

The experience and familiarity of the scorers with endometriosis in this study are clearly not representative of the average gynaecologist. 92% of the scorers spend~ more than 30% of their working hours looking after women with endometriosis. Therefore, these results cannot be extrapolated to all gynaecologists and trainees at all levels. Further studies are needed to assess the reliability of VNESS in real life in the hands of gynaecologists with all levels of expertise in this field who may come across patients with endometriosis.

The ICC analysis of the vaginal compartment may represent a statistical anomaly. The severity of the disease in the demonstrated videos may account for this score. If the study was performed again, a greater range of disease severity in this compartment may provide a more representative ICC value.
