# Supplementary material for: Visual-Numeric Endometriosis Scoring System (VNESS) for mapping surgical findings: A validation study
Source: Facts Views Vis Obgyn. 2024 Dec 27;16(4):429–39. doi: 10.52054/FVVO.16.4.051 (PMC11819782; doi:10.52054/FVVO.16.4.051)

Appendix 1 Example Screenshots of VNESS1 Video Clips Embedded in Survey. Top Left - Pouch of Douglas. Top Right - Right Pelvic Sidewall. Bottom Left - Left Adnexa. Bottom Right - Left Uterosacral Ligament.

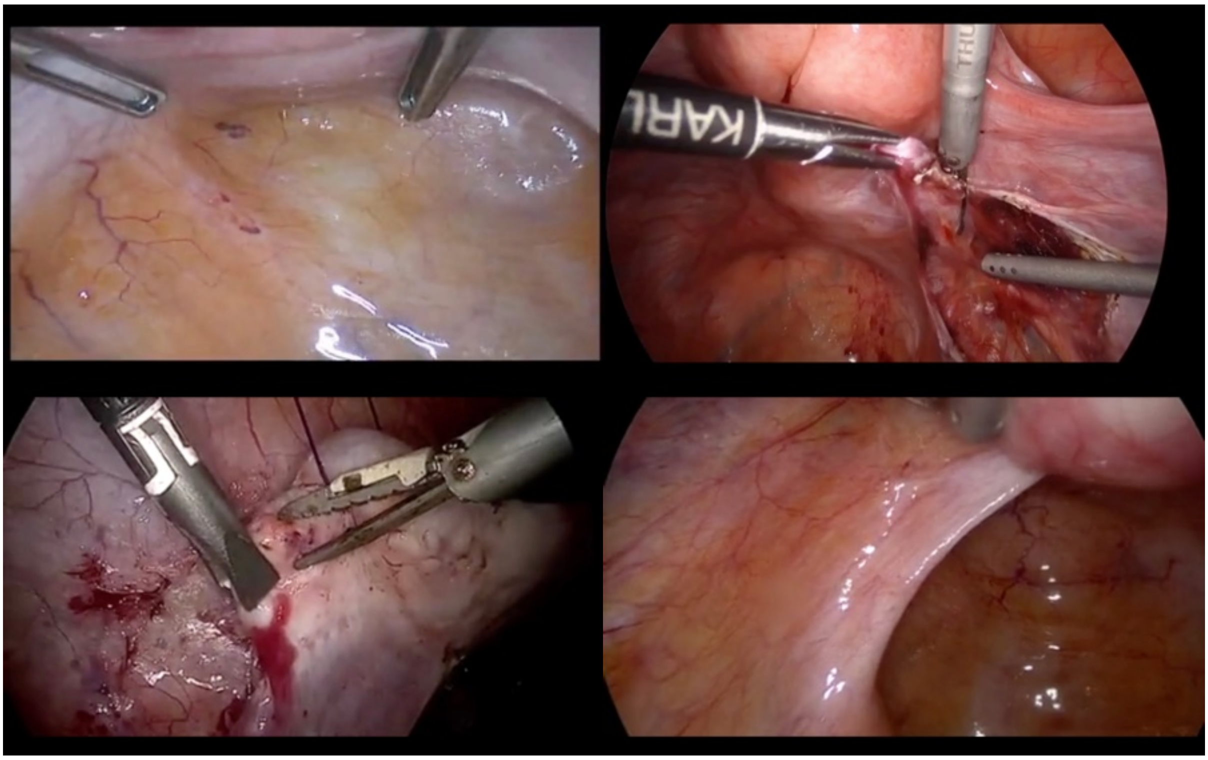

Appendix 2 Example Screenshots of VNESS2 Video Clips Embedded in Survey. Top Left - Pouch of Douglas. Top Right - Right Uterosacral Ligament. Bottom Left - Uterovesical Fold. Bottom Right - Left Adnexa (Endometrioma in an otherwise mobile ovary).

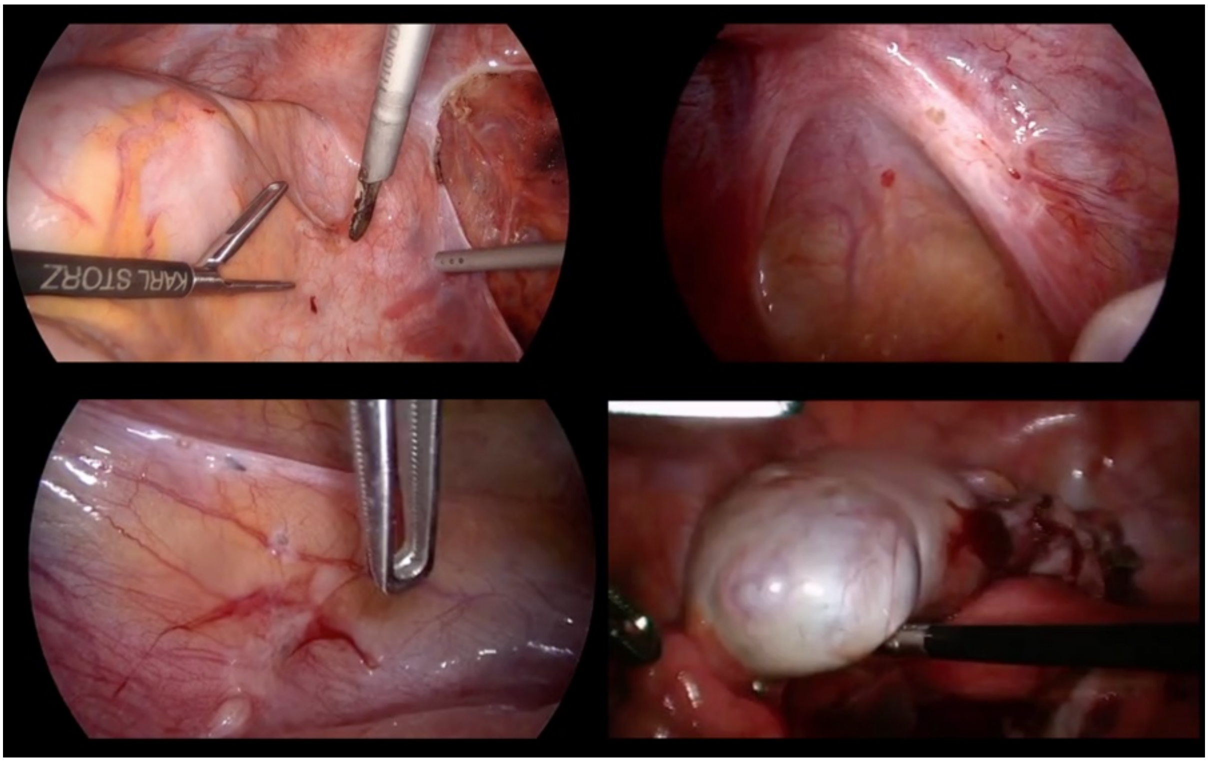

Appendix 3 Screenshots of VNESS3 Video Clips Embedded in Survey. Top Left - Pouch of Douglas. Top Right - Vagina. Bottom Left - Uterovesical Fold. Bottom Right - Left Adnexa (Endometrioma in a densely adherent ovary).

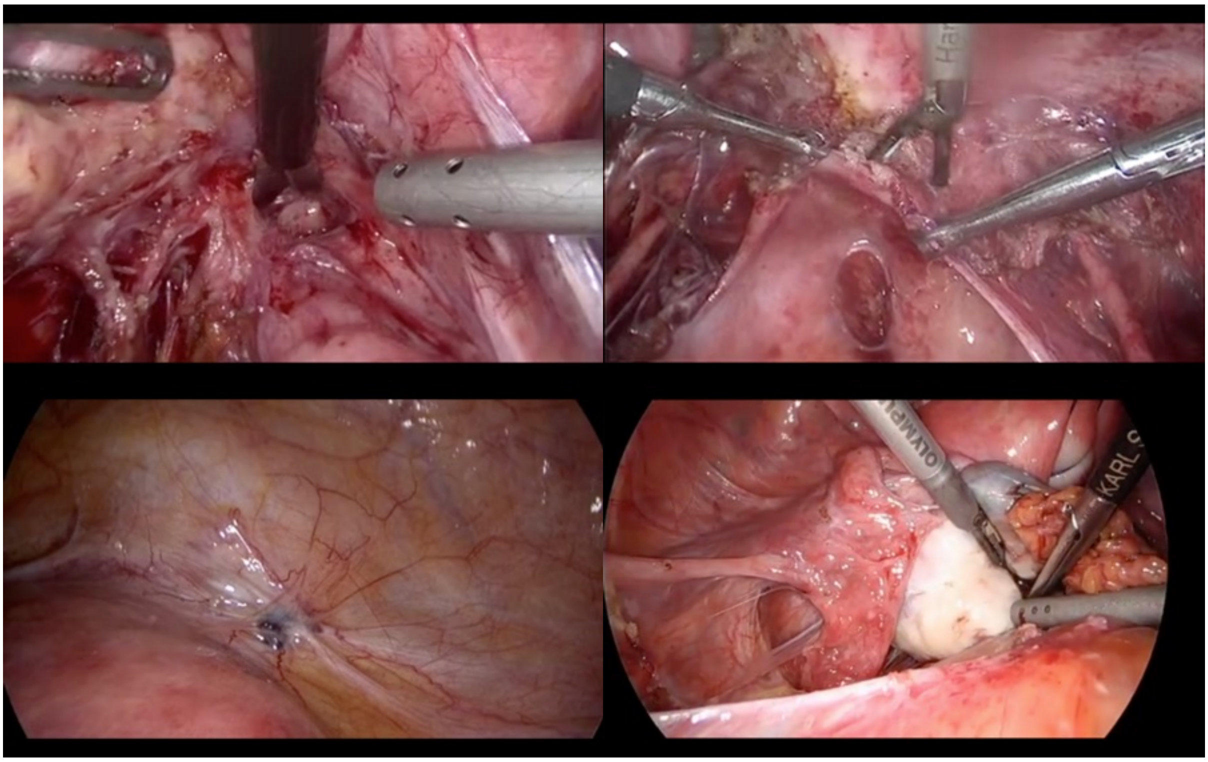

Appendix 4 Screenshots of VNESS 4 Video Clips Embedded in Survey. Top Left - Pouch of Douglas. Top Right - Vagina. Bottom Left - Uterovesical Fold. Bottom Right - Right Pelvic Sidewall (Ureteric Nodule).

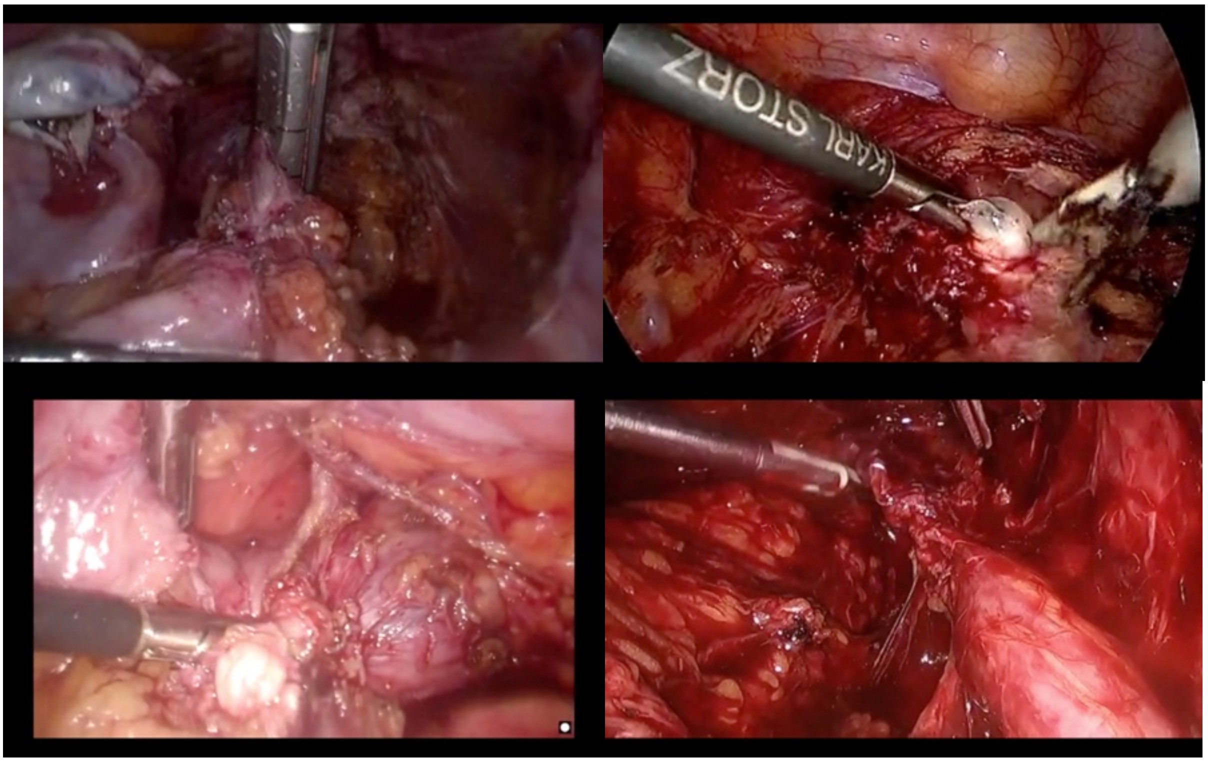

Appendix 5 Percentage of responders' monthly workload dedicated to the treatment of endometriosis

Percentage of Monthly workload spent for treatment of endometriosis	N (%)
0-30%	4 (8)
30-60%	13 (26)
60-90%	20 (40)
>90	13 (26)

Appendix 6 Number of Segmental Bowel Resection ever performed by the scorers as primary surgeon

Number of Segmental Bowel Resections ever	N (%)

performed as primary<br><br>Surgeon	
0	16 (32)
1-10	10 (20)
11-20	4 (8)
21-40	5 (10)
41-80	3 (6)
81-50	6 (12)
>150	6 (12)

Appendix 7 Agreement plot for adnexa

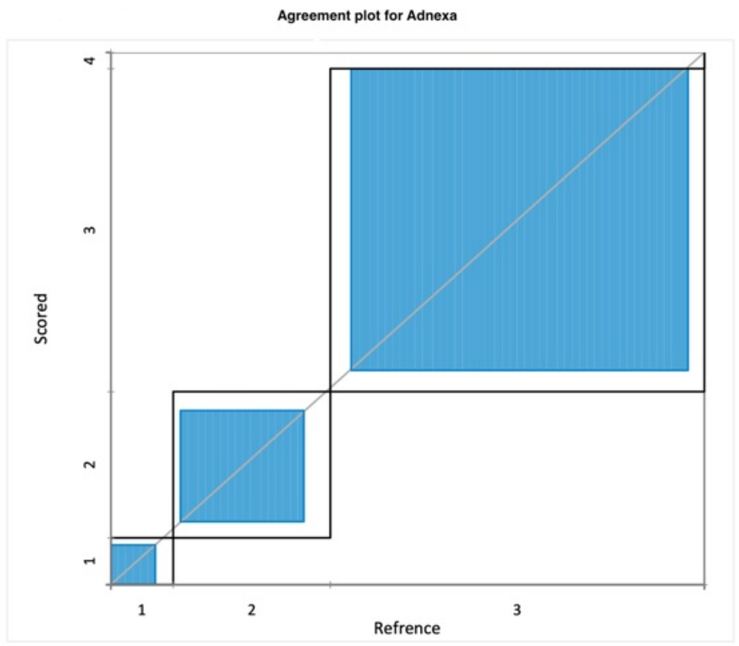

Appendix 8 Agreement plot for Pelvic side wall

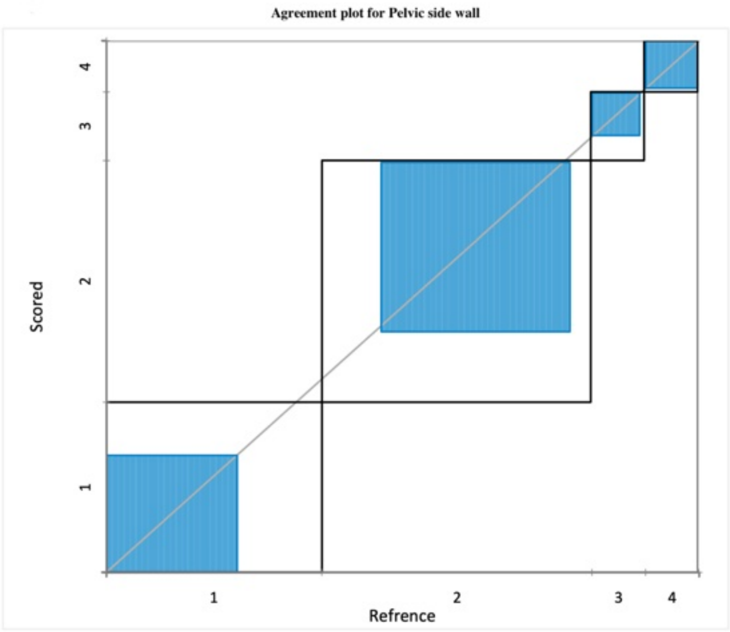

Appendix 9 Agreement plot for Uterosacral ligament

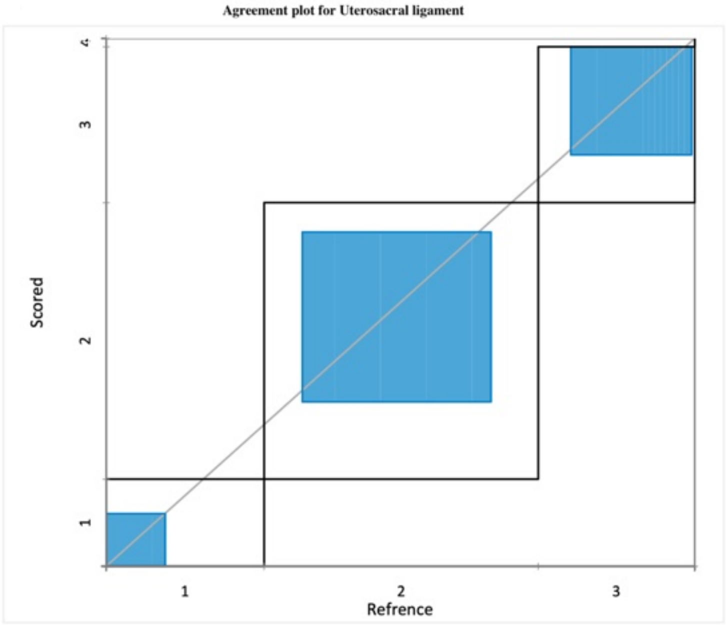

Appendix 10 Agreement plot for Uterovesical fold

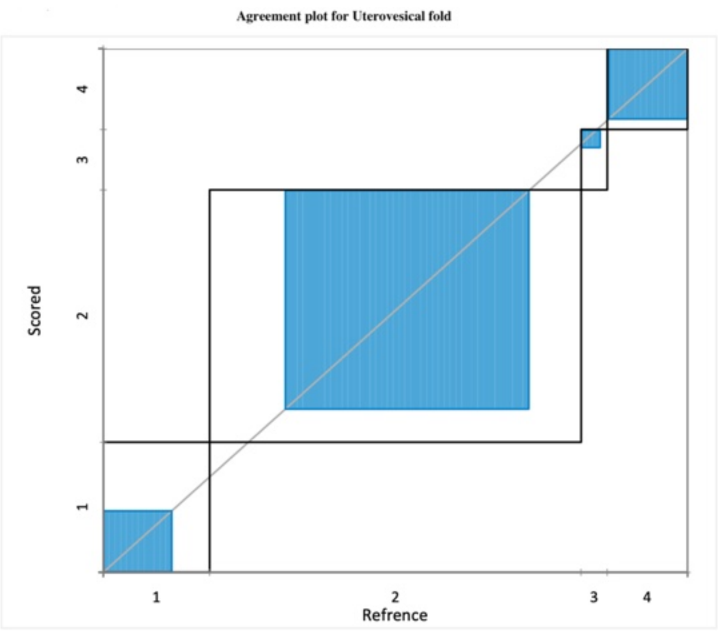

Appendix 11 Agreement plot for Vagina and rectovaginal septum

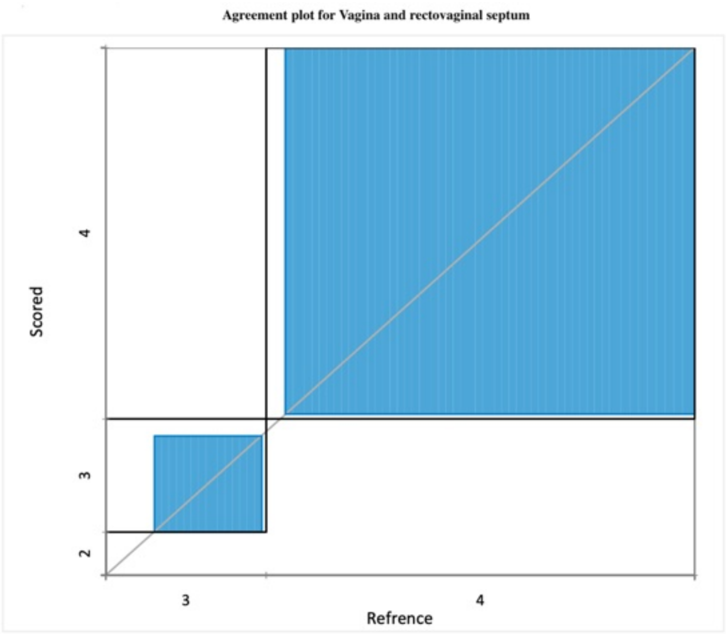

Appendix 12 Agreement plot for Rectum

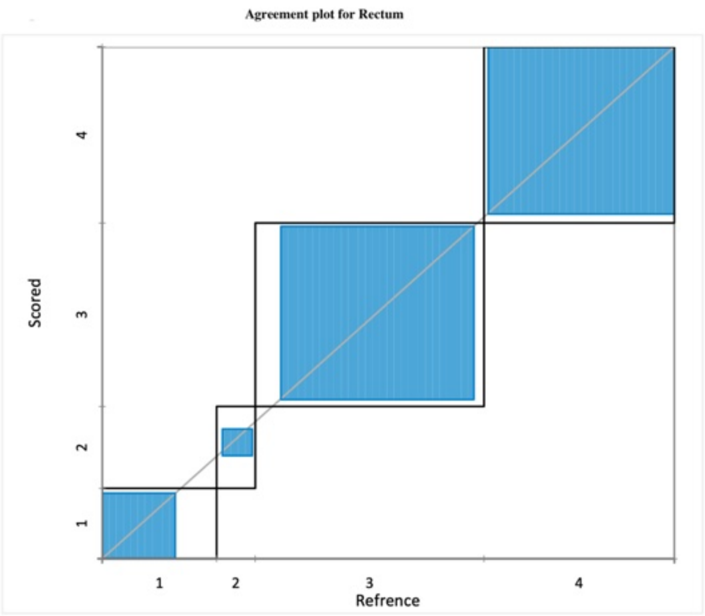

Appendix 13 Agreement plot overall for VNESS

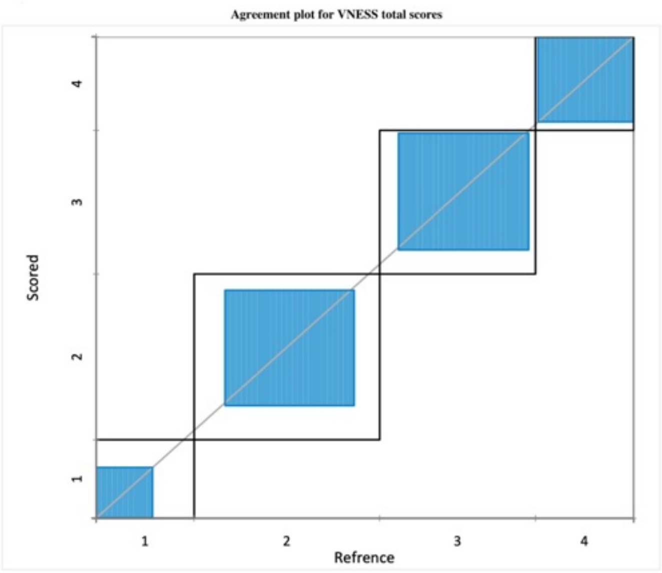

Supplement: Supplementary file 1 [file FVVinObGyn-16-429-apps.pdf]
